# Biopersistent Granular Dust and Chronic Obstructive Pulmonary Disease: A Systematic Review and Meta-Analysis

**DOI:** 10.1371/journal.pone.0080977

**Published:** 2013-11-20

**Authors:** Irene Brüske, Elisabeth Thiering, Joachim Heinrich, Katharina Huster, Dennis Nowak

**Affiliations:** 1 Institute of Epidemiology I, Helmholtz Zentrum München, German Research Center for Environmental Health, Munich, Germany; 2 Institute and Outpatient Clinic for Occupational, Social and Environmental Medicine, Clinical Centre, Ludwig Maximilian University, Munich, Germany; 3 Comprehensive Pneumology Center Munich (CPC-M), Member of the German Center for Lung Research, Munich, Germany; Pulmonary Research Institute at LungClinic Grosshansdorf, United States of America

## Abstract

**Objective:**

Applying a systematic review to identify studies eligible for meta-analysis of the association between occupational exposure to inorganic dust and the development of chronic obstructive pulmonary disease (COPD), and conducting a meta-analysis.

**Data Sources:**

Searches of PubMed and Embase for the time period 1970–2010 yielded 257 cross-sectional and longitudinal studies on people exposed to inorganic dust at the workplace with data on lung function. These studies were independently abstracted and evaluated by two authors; any disagreement was resolved by a third reviewer. Of 55 publications accepted for meta-analysis, 27 investigated the effects of occupational exposure to biopersistent granular dust (bg-dust).

**Methods:**

A random effects meta-analysis allowed us to provide an estimate of the average exposure effect on spirometric parameters presented in forest plots. Between-study heterogeneity was assessed by using I^2^ statistics, with I^2^>25% indicating significant heterogeneity. Publication bias was investigated by visual inspection of funnel plots. The influence of individual studies was assessed by dropping the respective study before pooling study-specific estimates.

**Results:**

The mean FEV1 of workers exposed to bg-dust was 160 ml lower or 5.7% less than predicted compared to workers with no/low exposure. The risk of an obstructive airway disease—defined as FEV1/FVC < 70%—increased by 7% per 1 mg· m^-3^ respirable bg-dust.

**Conclusion:**

Occupational inhalative exposure to bg-dust was associated with a statistically significant decreased FEV1 and FEV1/FVC revealing airway obstruction consistent with COPD.

## Introduction

Chronic obstructive pulmonary disease (COPD) is a common disease, and a substantive burden of COPD is attributable to risk factors other than smoking. Community-based studies from China [1], France [2], Italy [3,4], New Zealand [5], Norway [6], Spain [7,8], and the United States [9-13] have demonstrated increased relative risks for airway obstruction consistent with COPD associated with occupational exposure to vapour, dusts, gases, and fumes.

An official statement of the American Thoracic Society (ATS) concluded that an increased risk of chronic cough, lower FEV1 (forced expiratory volume in one second after full inspiration), and a lower FEV1/FVC (forced vital capacity) ratio was related to such occupational exposures [14,15]. The population attributable risk (PAR) of COPD from occupational exposure is estimated at 15-20% (16). But as these estimates are proportions, they depend on how causes other than vapour, dusts, gases, and fumes contribute to the development of COPD. The overall estimate of PAR of COPD due to occupational exposure may be misleading and a more quantitative approach seems preferable and is the objective of this meta-analysis.

Exposure to mineral dusts [16] especially in underground mining, such as gold-[17,18], coal-[19,20], and uranium mining [21] has been shown to contribute to the development of COPD, but not much is known about the impact of poorly soluble low-toxicity particles also referred to as biopersistent granular (bg) dust. We therefore conducted a systematic review and meta-analysis to quantitatively evaluate the association between occupational exposure to bg dust at the workplace and the development of COPD. 

## Materials and Methods

Following the PICOS criteria [22,23] **P**articipants, **I**ntervention, **C**omparison, **O**utcome, **S**tudy Design were defined in advance (see Checklist S1 in Appendix S1). We searched for epidemiological studies (cohort, case-control, and cross-sectional) of people exposed to bg dust at the workplace with measurements of exposure levels and spirometric measurements of lung function. As bg dust we considered: Portland cement, carbon black, soot, rubber, talcum, and occupational exposure during metal processing and mining (other than gold-, uranium- and coal mining). Not included as bg dust were environmental tobacco smoke and traffic related dust. We searched for studies in English and German between 1970 and 2010 in PubMed applying Medline (Medical Literature Analysis and Retrieval System Online) and in Embase (Excerpta Medica Database). The following MeSH-Terms were used: "occupational exposure” OR "air pollutants, occupational" AND "pulmonary disease, chronic obstructive” supplemented by the text fields “respiratory function tests" OR “respiratory function” OR “lung function” OR “pulmonary function”. Two investigators in our team (IB, KH) independently reviewed articles and extracted the following data in duplicate: first author and year of publication; study region and industry; study type; time and duration of the study or duration of the follow-up in cohort studies; number of exposed/unexposed subjects or cases and controls; sex- and age distribution of the study population, response rate; exposure assessment (interview, Job-Exposure-Matrix (JEM), type of dust measurement and average exposure to inhalable or respirable dust) outcome assessment (symptoms/physician diagnosis, spirometry and applied procedure for lung function measurements). Based on the abstracted protocol the validity of the study was independently assed and decision made jointly by the two reviewers (IB, KH); any disagreements were resolved by a third reviewer (DN). Studies were included in the systematic review and meta-analysis, if they fulfilled the following validity criteria: (1) transparent procedure of selection of study participants, no indication of selection bias; (2) response rate > 70% and < 100%; no response rate, or a response rate of 100%, which was considered as probably a post-hoc definition of the study population was not acceptable; (3) internal comparison with no/low exposed controls from the same company, or controls from a another company without exposure; (4) individual present or cumulative exposure preferably based on dust measurements (JEM was considered acceptable; company or questionnaire information was accepted only, if duration of exposure was also available); (5) COPD diagnosis according to obstructive signs in spirometry or physician-diagnosed (questionnaire information of symptoms was not considered as sufficient); (6) standardized pulmonary function test according to ATS/ERS criteria valid at the time of the study.

## Statistical Analysis

Assuming that the true effect of exposure to bg dust at the workplace differed from study to study, we conducted a random effects meta-analysis [24], which allowed us to provide an estimate of the average exposure effect. Between-study heterogeneity was assessed by using I^2^ statistics, with I^2^>40% representing moderate to considerable heterogeneity. Publication bias was investigated by visual inspection of funnel plots. The results of the meta-analysis in regard to specific lung function parameters of the cross-sectional and longitudinal analysis are presented in forest plots. The influence of individual studies was assessed by dropping the respective study before pooling study-specific estimates. 

The most important sign of airway obstruction - a reduced FEV1 and FEV1/FVC - was measured in most studies and used for the meta-analysis of cross-sectional analyses taking the difference of the mean FEV1 and FEV1/FVC among exposed study participants versus not/low exposed participants and also dependent on cumulative exposure to dust. FEV1 was measured in different units either in liters or in % predicted. FEV1% predicted is defined as FEV1 of the patient divided by the average FEV1 in the population for any person of similar age, height and sex. To combine both units [l] and [%] of FEV1 we calculated the standardized mean difference, which is the difference of the mean FEV1 of exposed and low/not exposed study participants divided by the common standard deviation. This measure is dimensionless. Different studies applied different methods to obtain the ratio FEV1/FVC. It was calculated as a ratio in liter, as a ratio in % predicted, and as a ratio of two predicted values. Only the standardized mean difference of the ratio was used as a common estimate of FEV1/FVC for meta-analysis. Studies either adjusted or stratified for smoking status. In the latter case the results for smokers and nonsmokers were integrated separately into the meta-analysis. For some studies it was possible to perform a meta-analysis of the risk of obstructive airway disease by FEV1 and/or FEV1/FVC per 1mg/m^3^ of bg dust. And for some longitudinal studies the annual decline of FEV1 among exposed and unexposed study participants could be compared and integrated in the meta-analysis. 

## Results

**Figure 1:**
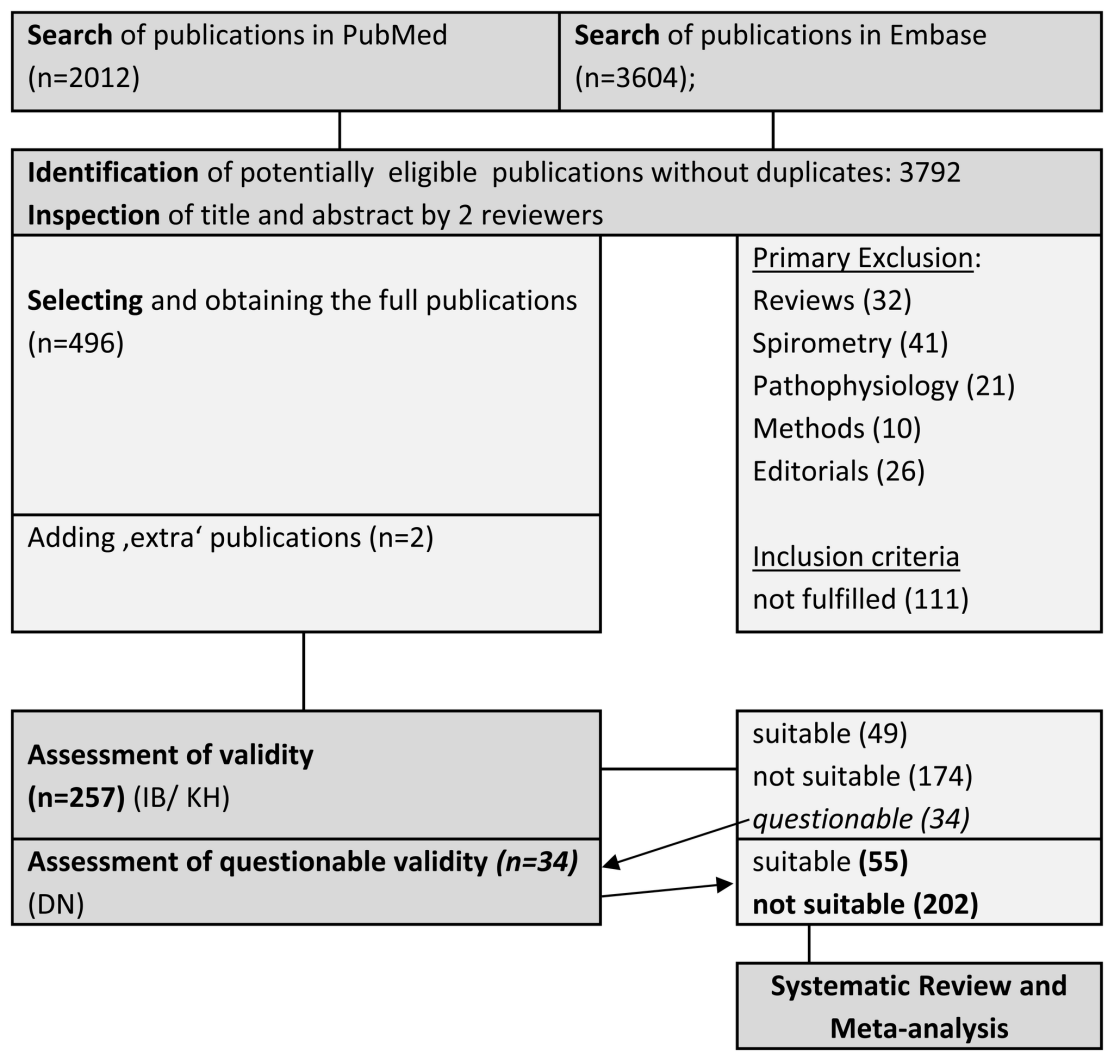
Flow chart of study selection for Systematic Review and Meta-Analysis.

2012 publications were identified in PubMed and as well as 3604 publications in Embase. Without duplicates 3792 publications were potentially eligible. Title and abstract were screened by two investigators (IB, DN). Two German publications were added manually, one was a recent publication [21] outside the defined time frame and one [25] was a large longitudinal investigation among construction workers, which was published in a journal not listed in Embase. For details of the selection process see Figure 1.

After excluding studies that did not fulfill the inclusion criteria 257 publications were reviewed with data abstraction, 55 fulfilled the validity criteria and were accepted for review and meta-analysis. 27 publications [26-52] thereof investigated the effects of occupational exposure to bg dust (see Appendix S1, Table 1). Not all of these 27 studies could actually be included in the meta-analysis, some because of choosing rarely used endpoints [40,41,50-52], some because of not providing a standard error [28,37], for more details see see Appendix S1, Table 1. 

**Table 1:** Review of the 27 selected studies on biopersistent granular dust and airway obstruction by first author [26-52].

**First author/ year**	**Country**	**Bg dust exposure**		**Industry/ measurements of exposure to inhalable and respirable dust [mg·m^-^**³]	**parameter chosen for meta-analysis (MA)**
		yes	no		
Abrons 1988	USA	2607	729	Portland cement/ inhalable dust (GM*) 2.9 [mg·m^-^³], respirable dust (GM) 0.57 [mg·m^-^³] high vs. zero	FEV1
AbuDhaise 1997	Jordan	99	129	Portland cement/ 3 levels of exposure to respirable dust (GM) 0.5/1.6/3.9 [mg·m^-^³] (high vs. low exposure)	FEV1, FEV1/FVC
Beach 2001	Australia	572	79	Bauxite open pit mining/ inhalable dust (GM) 0.44 - 0.65 mg·m^-^³, respirable dust (GM) 0.14-0.26 mg·m^-^³, Quartiles <2.5 /2.5-6.0/ 6.1-10/>10 mg·m^-3^·years	FEV1, FEV1/FVC – without SE, not suited for MA
Boojar 2002	Iran	141	65	Manganese underground mining/ total dust (manganese content), cumulative respirable dust (JEM) [mg·m^-^³·years]	FEV1, FEV1/FVC
Chan-Yeung 1989	Canada	164	308	aluminium smelter ‚High‘, if >50% working hours in the potroom	FEV1
		75	115		Decrease of FEV1 [ml ·year^-1^]
Chen 2006	Taiwan	394	309	Steelworkers/ inhalable dust (AM) 3.55 mg·m^-^³, follow-up 1.90 mg·m^-^³	FEV1, FEV1/FVC
Fell 2003	Norway	119	50	Portland cement	Part of Nordby 2011 not included in MA
Fine 1976; Teil III	USA	65	141	Rubber workers/ respirable dust 1.05-3.00 mg·m^-3^ high vs. zero (cumulative dust years)	FEV1, FEV1/FVC
Fine 1976; Teil IV	USA	91	141	Talc workers/ respirable dust 0.47-3.55 mg·m^-3^ high vs. zero (cumulative dust years)	
Gardiner 1993	Europe	509	277	Carbon black/ inhalable dust max. 1.60 mg·m^-3^ and respirable dust >0.45 mg·m^-3^ in 5 exposure groups, JEM cumulative exposure [mg·m^-^³·months]	FEV1
Gardiner 2001	Europe	Phase 2: 2324	Phase 3: 1994	Phase 2, cumulative 263.2 mg·m^-^³·months; current exposure 0.77 mg·m^-^³Phase 3, cumulative 245.9 mg·m^-^³· months; current exposure 0.57 mg·m^-^³	Decrease of FEV1, FVC, FEV1/FVC per 1 mg·m^-3^
Harber 2003	USA	416	236	Carbon black / total, inhalable, and respirable dust, current and cumulative (JEM), classification into pentile groups	FEV1, decrease of FEV1 per mg·m^-3^·years without SE, not suited for MA
Huvinen 1996	Finland	36	93	Stainless steel production/ Cr^+3^, Fe^+2^Cr_2_O_4_ (Chromit), „average dust concentration“ 1 - 1.8 mg·m^-3^	FEV1, FEV1/FVC
Johnsen 2008	Norway	1812	532	Smelter / inhalable dust and respirable dust according to working area	FEV1
Kongerud 1990	Norway	1760	0	Aluminium potroom workers/ total dust (median) 3.25 mg ·m^-^³ ; OR for obstruction according to duration of employment	FEV1 minus predicted divided by residual standard deviation – not suited for MA
Kuo 1999	Taiwan	291	105	Foundry workers/ respirable dust 1.89 mg·m^-3^ (molding), 2.76 mg·m^-3^ (furnace), 2.07 mg·m^-3^ (after- processing)	FEV1, FEV1/FVC,
		308	112		decrease of FEV1 [ml·year^-1^] without SE, not suited for MA
Lotz 2008	Germany	1.Study A: 402 B: 438	0	Underground potash mining Company A: respirable dust (AM) 1.96 mg·m^-^³ and inhalable dust (AM) 14.2 mg·m^-^³ ; cumulative respirable dust 613 mg·m^-^³·months; cumulative inhalable dust 4419 mg·m^-^³·months; Company B: respirable dust (AM) 0.88 mg·m^-^³ and inhalable dust (AM) 5.65 mg·m^-^³ ; cumulative respirable dust 165 mg·m^-^³·months, cumulative inhalable dust 1060 mg·m^-^³·months	
		2.Study A: 290 B: 278	0		Decrease of FEV1 per 1·mg·m^-^³
Meijer 1998	Netherland	70	69	Rubber workers/ inhalable dust (AM) 2 mg·m^-^³ cumulative (JEM) 32.5 mg·m^-^³·years	FEV1, FEV1/FVC
		70	69		decrease of FEV1 per mg·m^-3^·year
Mwaiselage 2004	Tanzania	115	102	Portland cement/ inhalable dust (GM) 10.6 mg·m^-^³, cumulative dust (GM) 69.1 mg·m^-^³·years (high vs. low exposure)	FEV1, FEV1/FVC
		115	102		decrease of FEV1 per 1 mg·m^-3^·year
Neghab 2007	Iran	88	80	Portland cement/ inhalable dust (AM**) 53.4 mg·m^-^³ respirable dust (AM) 26 mg·m^-^³	FEV1, FEV1/FVC
Neghab 2007	Iran	97	110	Rubber industry/ inhalable dust (AM) 41.8 mg·m^-^³ and respirable dust (AM) 19.8 mg·m^-^³	FEV1, FEV1/FVC
Nordby 2011	Europe	1406	629	Portland cement/ inhalable dust (GM) 0.85 mg·m^-^³, classification by means of a JEM into quartiles <0.49/0.49-1.08/1.09—1.73/>1.74 [mg·m^-3^ ]	FEV1, FEV1/FVC and OR FEV1/FVC < 70% per 1 mg·m^-3^ inhalable dust
Selden 2001	Sweden	34	61	Dolomite mining/ total dust (median): 2.8 mg·m^-3^	FEV1
Soyseth 2011	Norway	3392	532	Smelter/ inhalable dust and respirable dust according to working area	OR for FEV1/FVC <70% per 1 mg·m^-3^
Townsend 1985	USA	1146	0	Aluminium production/ cumulative total dust (JEM) [mg·m^-3^ years] comparing < 100 mg·m^-3^ years and ≥ 100 mg·m^-3^ ·years for three categories of duration <10 years, 10-19 years, ≥ 20 years	FEV1 minus KNUDSON predicted FEV1 – not suited for MA
Wang 1996	USA	475	0	Steel workers No dust measurements, exposed years in „dusty areas“	Number of exposed years only, -not suited for MA
Wild 1995	France	138	55	Talc producing/ respirable dust (GM) 1.87 mg·m^-^³ cumulative exposure according to JEM mg·m^-^³·years	Standardized residuals for FVC and FEV1 – not suited for MA

For the studies with two rows, the 1st row is related to the cross-sectional analysis and the 2nd row to the longitudinal analysis

*GM: geometric mean

**AM: arithmetic mean

***JEM: job-exposure matrix

Short summary of the review: Early studies from the US (26) and Jordan (27) showed no detrimental effect of dust exposure on lung function in cement workers. But, later studies with a higher dust exposure of cement workers from Tanzania (44) and Iran (45) showed a strong association with impaired lung function. Especially, a new large and prospectively designed study of the European Cement Association (47) with 4265 exposed participants was very informative. All studies related to carbon black/soot/rubber/talcum (33–37,43,46,52) showed adverse effects of dust exposure on lung function with stronger effects for early studies with high dust exposure compared to late studies with low exposure. The evidence from studies with metal workers in the aluminum, iron and steel industry was inconsistent (30,31,38–41,49,51). Negative or only weakly positive (and not statistically significant) impact of dust exposure on lung function was seen in studies from Canada (30), Finland (38) and the US (50). Exposure to dust in the mining industry varied a lot in regard to type, composition and intensity of dust exposure. While workers in a large open pit mine of Bauxite in Australia (28) were only low exposed and showed no lung function abnormalities, there was a high dust exposure in manganese mining underground in Iran (29). Here, spirometry showed a significant decrease of FEV1 and FVC of exposed workers compared to non-exposed workers with a stronger effect in smokers (29). A longitudinal study (42) performed in potash mining underground in Germany showed also a significant decrease in lung function over time.

**Figure 2:**
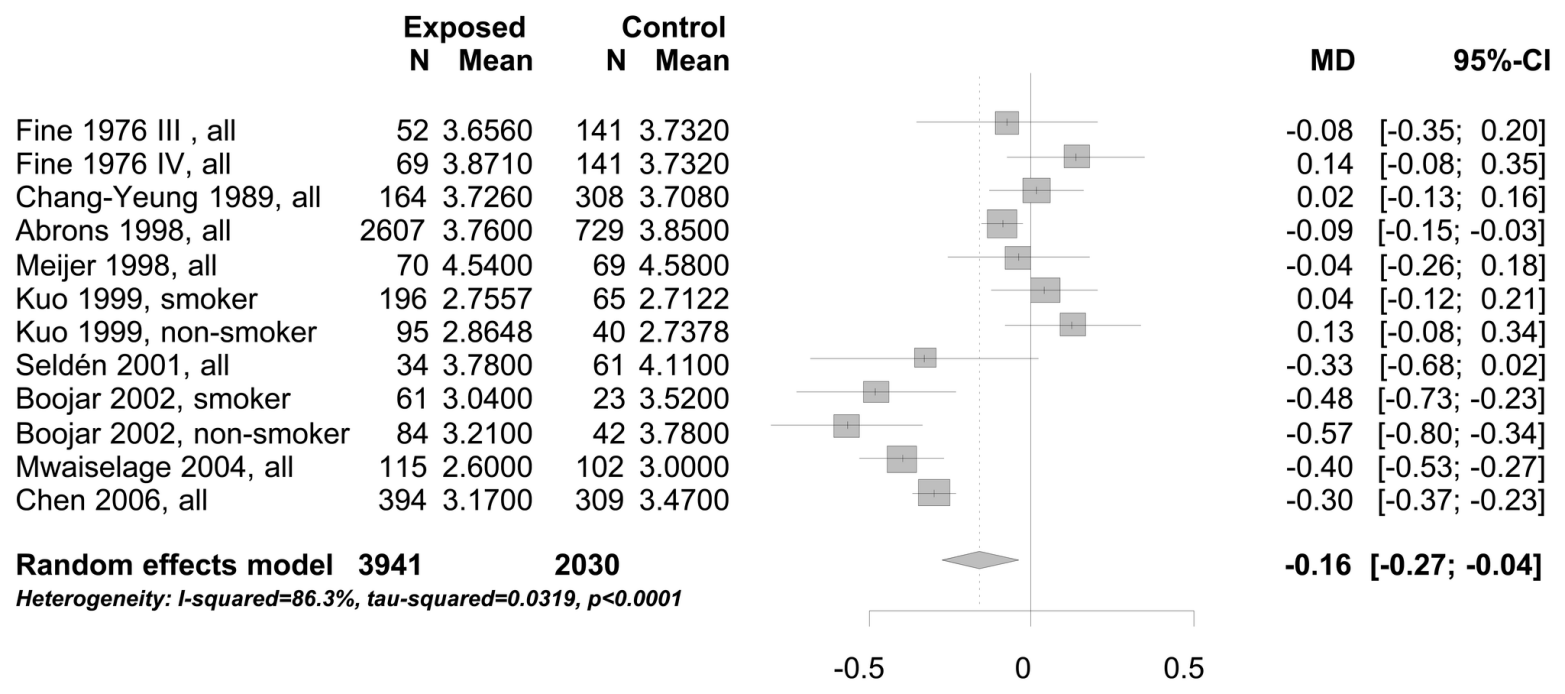
Mean difference (MD) of FEV1 [liter] between study participants exposed to bg dust at the workplace and no/low exposed participants.

**Figure 3:**
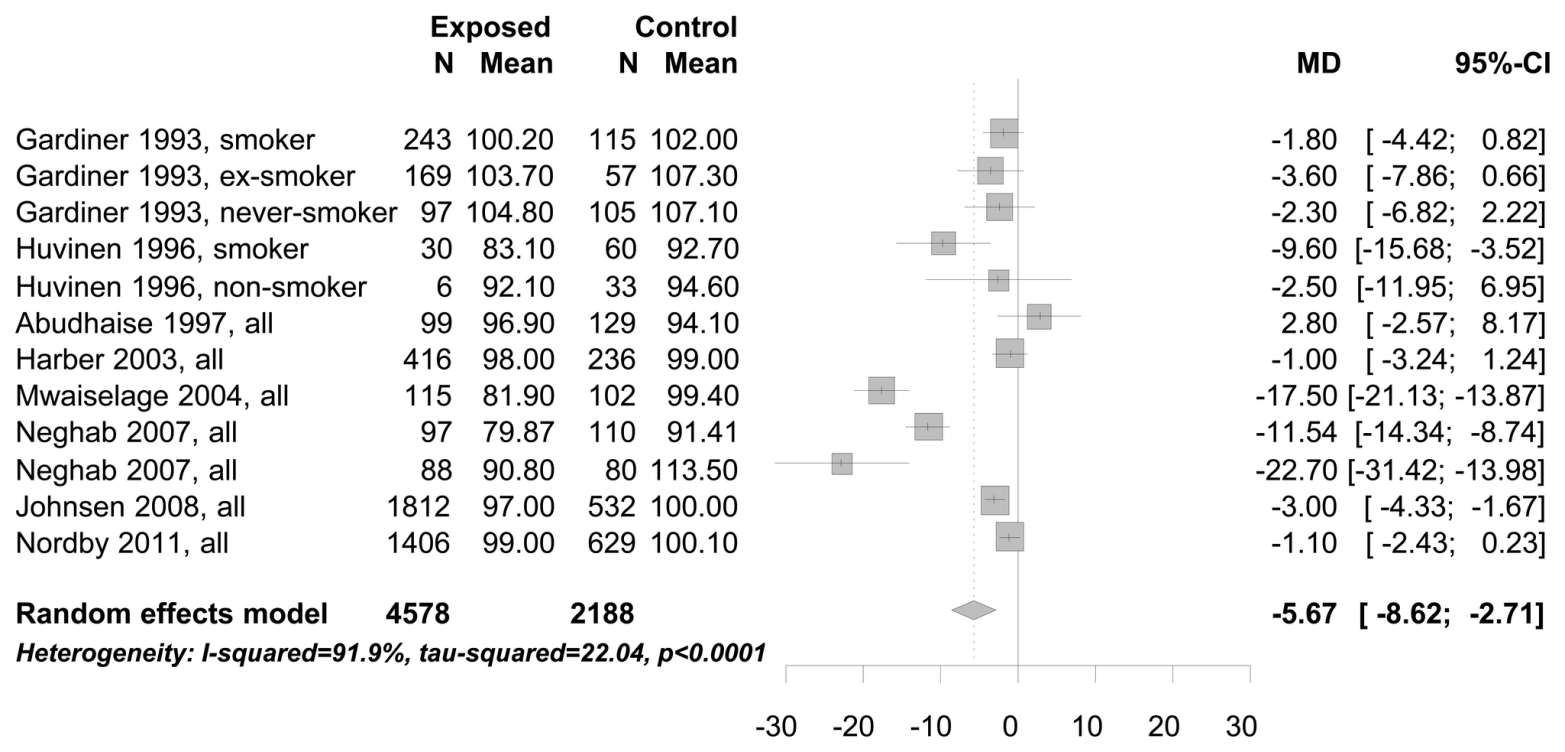
Mean difference (MD) of FEV1 in % predicted between study participants exposed to bg dust at the workplace and no/low exposed participants.

**Figure 4:**
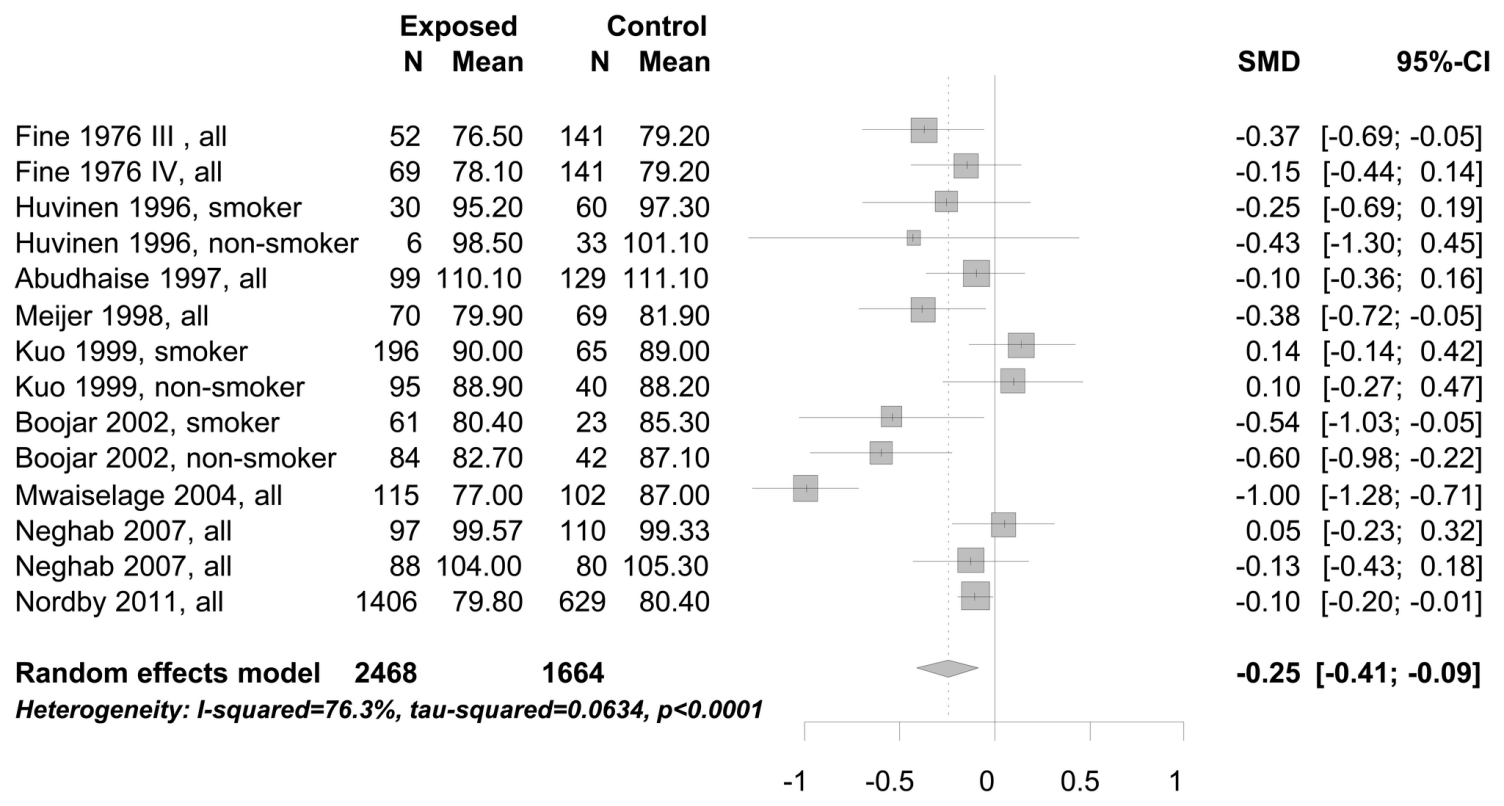
Standardized mean difference of the ratio FEV1/FVC between study participants exposed to bg dust at the work place and low/no exposed participants.

In the meta-analysis of cross-sectional study results we combined studies with endpoints measured in the same units: FEV1 measured in liter (see Figure 2), and FEV1 measured in % predicted (see Figure 3). The mean FEV1 of workers exposed to bg dust was 160 ml (95% CI: 40-270 ml) less than compared to workers with no/low exposure (see Figure 2). Comparing the mean FEV1 not in absolute measures [liter], but in % predicted, it was 5.7% (95% CI: 2.71-8.62%) lower for workers exposed to bg dust (see Figure 3). There was also a decrease of FEV1, taking the standardized mean difference between exposed and no/low exposed workers into the meta-analysis (results not shown). Different studies applied different methods to obtain the ratio FEV1/FVC. Only the standardized mean difference of the ratio could be used as a common estimate of FEV1/FVC for meta-analysis. The mean difference of the ratio FEV1/FVC between study participants exposed to bg dust at the work place and low/no exposed participants was significantly decreased -0.25 (95% CI:-0.09 to -0.41)(see Figure 4).

**Figure 5:**
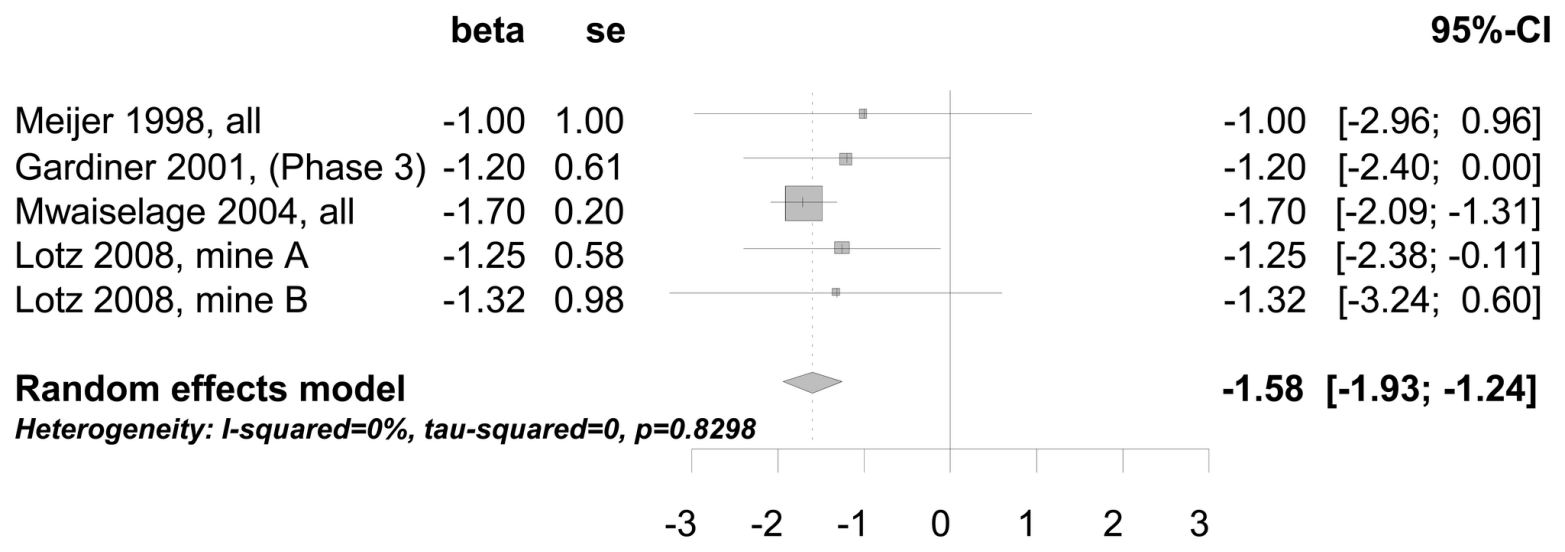
Decrease of FEV1 (ml) in relation to the cumulative exposure to bg dust at the workplace (mg·m^-3^·years).

In the meta-analysis of longitudinal study results two studies [30,53] showed a mean annual decline of FEV1 of 6.3 ml higher for bg dust exposed participants compared to low/no exposed participants (results not shown). Using studies [36,42-44] with a cumulative measure for the decline of lung function related to bg dust exposure [mg·m^-3^·years], the meta-analysis showed a decline of FEV1 of 1.6 ml per 1 mg·m^-3^·years (meta-analysis 1.58 ml (95% CI: 1.24-1.93ml)) (see Figure **5**). And finally, applying the GOLD criteria two studies reported an increased odds ratio for COPD (FEV1/FVC < 70%) of 1.06 [47] and 1.07 [49] related to the increase of 1 mg·m^-3^ bg dust. The visual inspection of funnel plots gave no indication of publication bias, as large studies tended to be near the average and there were also small studies that reported null findings (see Figures S1-4 in Appendix S1).

## Discussion

The meta-analysis revealed a strong heterogeneity between the studies which had to be expected considering the variant exposure conditions at the workplace in different countries from Europe and abroad over such a long time span. The results from the analysis of highly exposed workers indicated a stronger effect than in all workers combined [44,46]. Nevertheless, dropping the respective studies before pooling study-specific estimates had only a minor impact on the results of the meta-analysis and no impact on the statistical significance. 

Inhalation of mineral dust such as quartz and asbestos fibers will induce fibrotic changes of the lung parenchyma accompanied by restrictive spirometric changes, such as a reduced FVC. No such findings were reported for biopersistent granular dust. The meta-analysis of cross-sectional studies showed an association of bg dust only with obstructive symptoms in the spirometry. The mean FEV1 of workers exposed to bg dust was 160 ml lower or 5.7% less than predicted compared to workers with no/low exposure. Whatever measure for airway obstruction was used the reduction of FEV1 or FEV1/FVC was always statistically significant. However, this is probably an underestimate of the true effect of bg dust exposure, as subjects with impaired lung function are more likely to quit their jobs and will therefore not be available as study participants [54]. This selection bias will be even stronger, when investigating an actual obstructive limitation, such as FEV1/FVC < 70% according to the GOLD criteria. Workers with such an impairment plus respiratory symptoms will probably not stay in the workforce. Nevertheless, the risk of an obstructive airway disease - defined as FEV1/FVC < 70% - increased by 7% per 1 mg· m^-3^ bg dust [47,49].

If the inhalation of bg dust causes COPD, the exposure should be associated with an accelerated decline in lung function, which cannot be detected in a cross-sectional study design [55]. A longitudinal design including repeated spirometries in each person during a period of several years is needed. Two studies [30,53] showed a mean annual decline of FEV1 of 6.3 ml higher in bg dust exposed participants compared to low/no exposed participants. The observed effect (adjusted for age and smoking) was quite similar to the 7-8 ml reported before [56,57] and appears to be rather small compared to the normal age-related reduction of FEV1 (15-25 ml/year) and the decrease due to smoking (60-80 ml/year) [58]. A few studies provided data for the decline of FEV1 related to a cumulative dust concentration at the workplace [35,42-44]. These studies showed very consistently a decrease of 1.6 ml (95% CI: 1.24-1.93ml) per 1mg·m^-3^·years.

As the loss of FEV1 per year is typically small, it tends to be hidden by measurement variability and will become obvious only in longer follow-up periods. Whereas Wang et al. [59] consider a decrease of FEV1 > 8% or 330ml per year at the workplace as probably pathological, other authors (Hnizdo et al. 2006; Hnizdo et al. 2007) have suggested a method with higher sensitivity to estimate the „longitudinal limits of normal decline“ . According to the authors, a decrease of more than 60ml per year should be suggestive of an increasing airway obstruction.

Aiming at a quantitative assessment of the association between occupational exposures to bg dust at the workplace and the development of obstructive symptoms in spirometry, the requirements for a study to be included in the meta-analysis were very specific and led to a remarkable drop between studies identified in the systematic review and those finally included in the meta-analysis. From this follows that the studies included in the meta-analysis cannot claim to be representative of all studies on the subject, but only for those with quantitative data on bg dust exposure at the workplace and lung function measurements. For a more general overview refer to [60-64].

At present, COPD as a compensable occupational disease is included in two international lists of occupational diseases, one proposed by the International Labour Organization (ILO) [65], and the other established by the European Commission [66]. Both are only recommendatory in character; most EU-member states have their own lists, which are comparable just in some parts [67]. In Germany, COPD or emphysema due to underground bituminous coal mining with a cumulative exposure to respirable dust exceeding 100 (mg/m^3^) x years is already part of the list of recognized occupational diseases entitled to compensation. Other jobs with comparable exposure levels to mineral dust or bg dust are presently not covered by the definition.

In summary, this meta-analysis shows a consistent decline of FEV1 of about 1.6 ml in regard to a cumulative bg dust concentration at the workplace of 1mg·m^-3^·years . Occupational exposure to bg dust was associated with a statistically significantly decreased FEV1 and FEV1/FVC revealing an airway obstruction consistent with COPD. The observed limitation of pulmonary function in workers exposed to bg dust probably underestimated the true effect, since both cross-sectional and longitudinal studies in the workforce are often limited to a ‘‘survivor’’ population because of the inability to monitor workers who leave their jobs. 

## Supporting Information

Appendix S1 **Supporting files**.
Figure S1, Funnelplot for Figure 2 Mean difference (MD) of FEV1 [liter] between study participants exposed to bg dust at the workplace and no/low exposed participants. Figure S2, Funnelplot for Figure 3 Mean difference (MD) of FEV1 in % predicted between study participants exposed to bg dust at the workplace and no/low exposed participants. Figure S3, Funnelplot for Figure 4 Standardized mean difference of the ratio FEV1/FVC between study participants exposed to bg dust at the work place and low/no exposed participants. Figure S4, Funnelplot for Figure 5 Decrease of FEV1 (ml) in relation to the cumulative exposure to bg dust at the workplace (1 mg·m^-3^·years). Checklist S1, PRISMA 2009 Checklist.
(DOC)
