# Supplementary material for: Biopersistent Granular Dust and Chronic Obstructive Pulmonary Disease: A Systematic Review and Meta-Analysis
Source: PLoS One. 2013 Nov 20;8(11):e80977. doi: 10.1371/journal.pone.0080977 (PMC3835577; doi:10.1371/journal.pone.0080977)
Supplement: Appendix S1 — Supporting files. Figure S1, Funnelplot for Figure 2 Mean difference (MD) of FEV1 [liter] between study participants exposed to bg dust at the workplace and no/low exposed participants. Figure S2, Funnelplot for Figure 3 Mean difference (MD) of FEV1 in % predicted between study participants exposed to bg dust at the workplace and no/low exposed participants. Figure S3, Funnelplot for Figure 4 Standardized mean difference of the ratio FEV1/FVC between study participants exposed to bg dust at the work place and low/no exposed participants. Figure S4, Funnelplot for Figure 5 Decrease of FEV1 (ml) in relation to the cumulative exposure to bg dust at the workplace (1 mg·m-3·years). Checklist S1, PRISMA 2009 Checklist. (DOC) [file pone.0080977.s001.doc]

Appendix S1


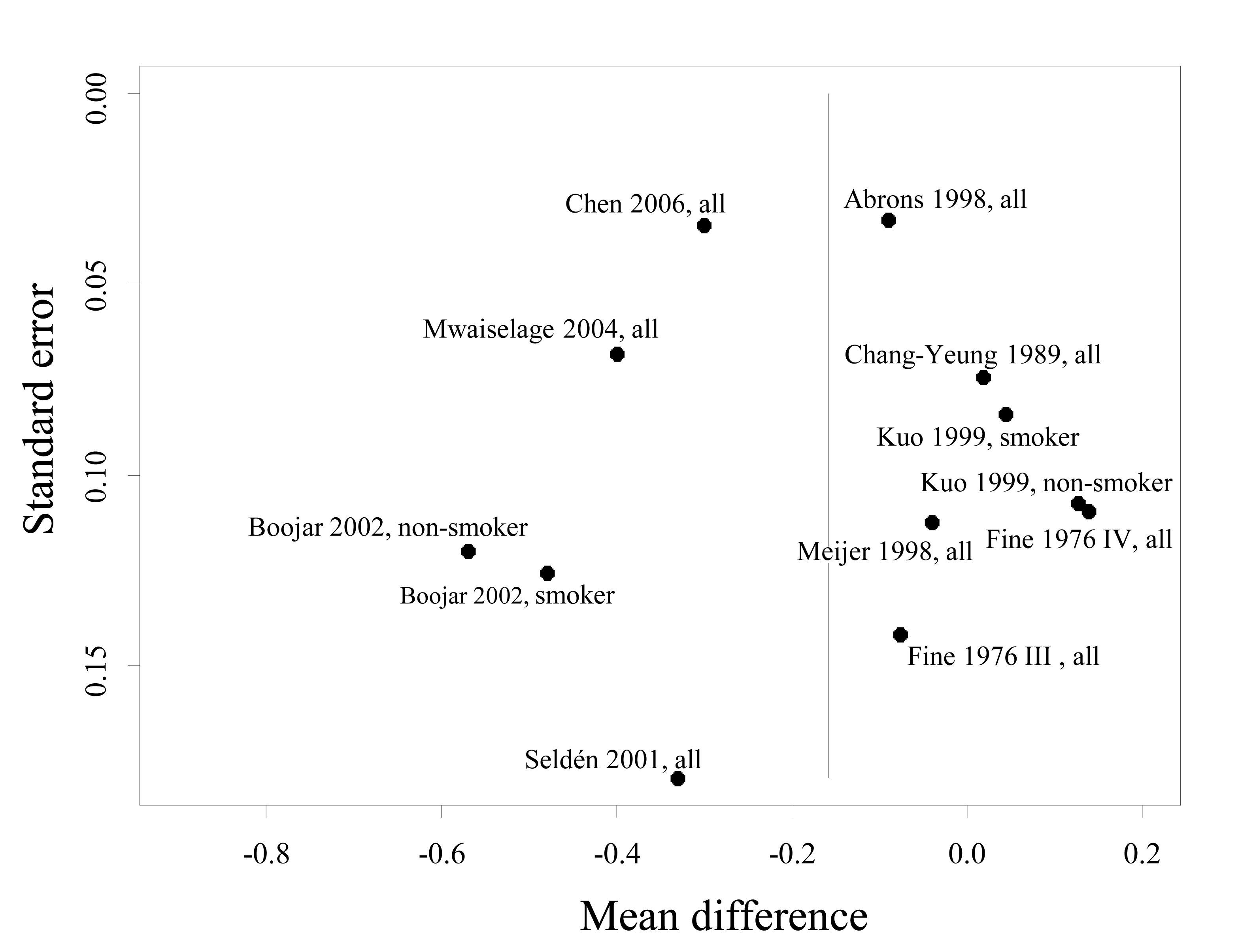


Figure S1 Funnelplot for figure 2 Mean difference (MD) of FEV1 [liter] between study participants exposed to bg dust at the workplace and no/low exposed participants

**
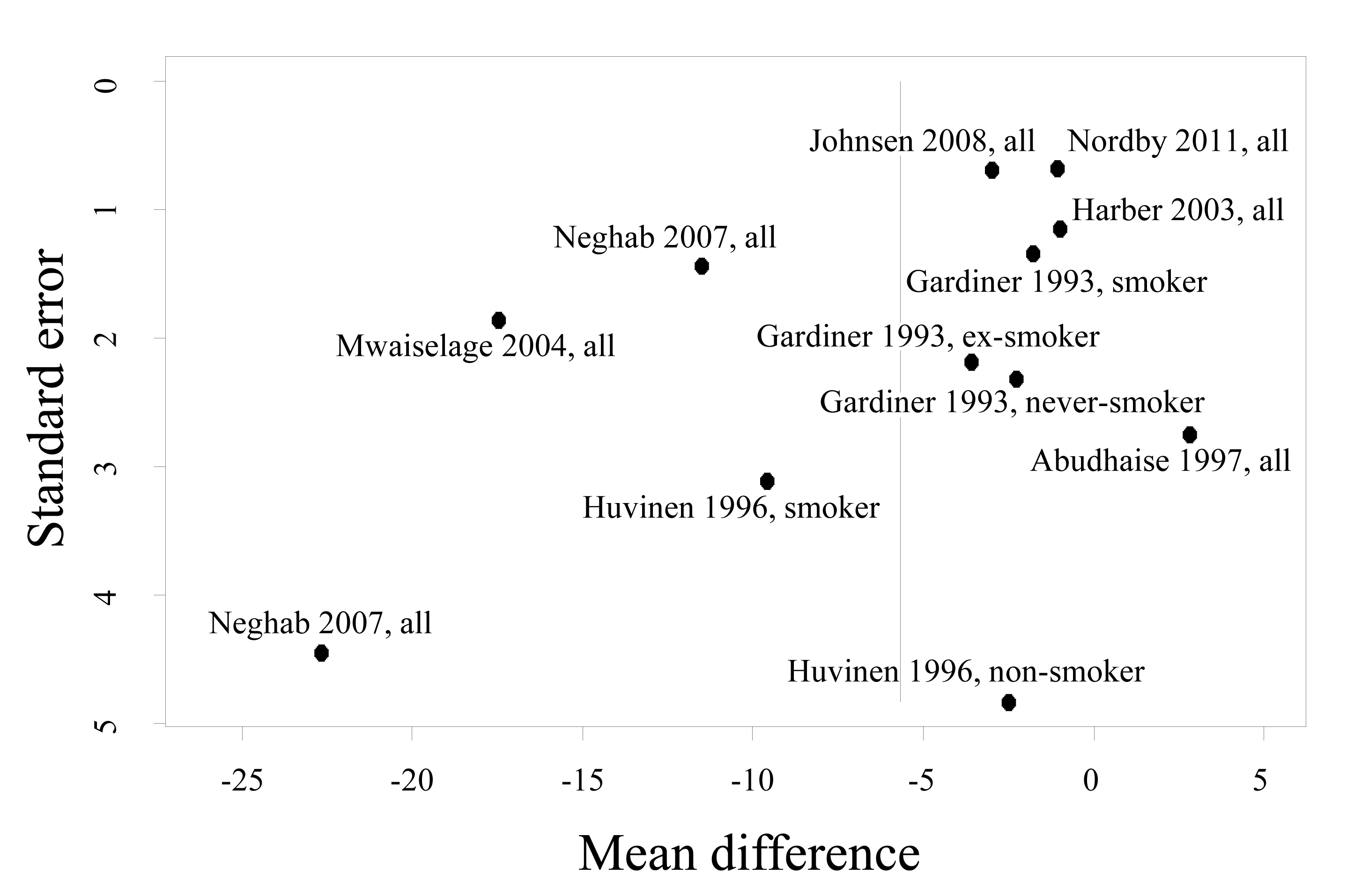
**

**Figure S2 Funnelplot for figure 3** Mean difference (MD) of FEV1 in % predicted between study participants exposed to bg dust at the workplace and no/low exposed participants


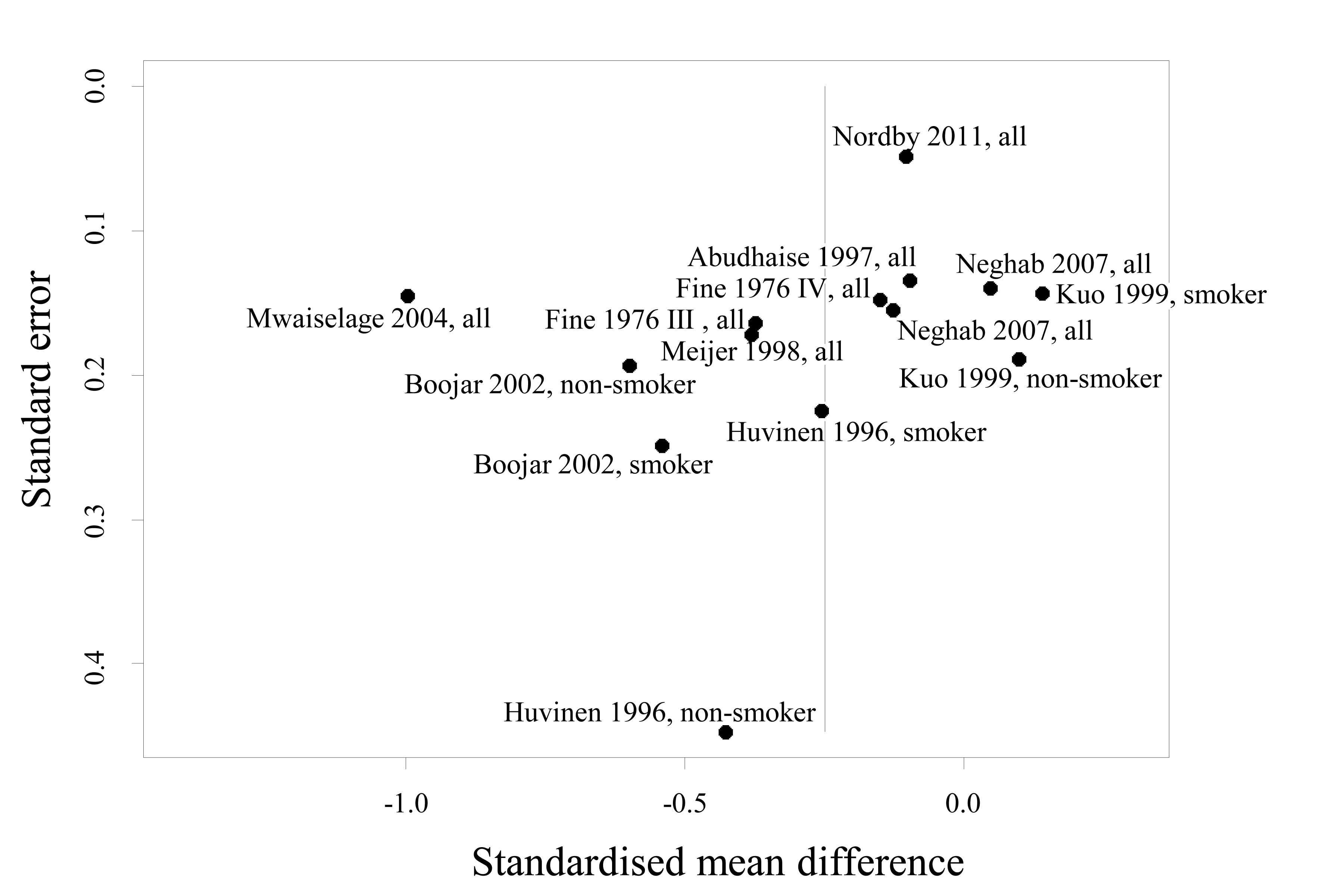


**Figure S3 Funnelplot for figure 4** Standardized mean difference of the ratio FEV1/FVC between study participants exposed to bg dust at the work place and low/no exposed participants

**
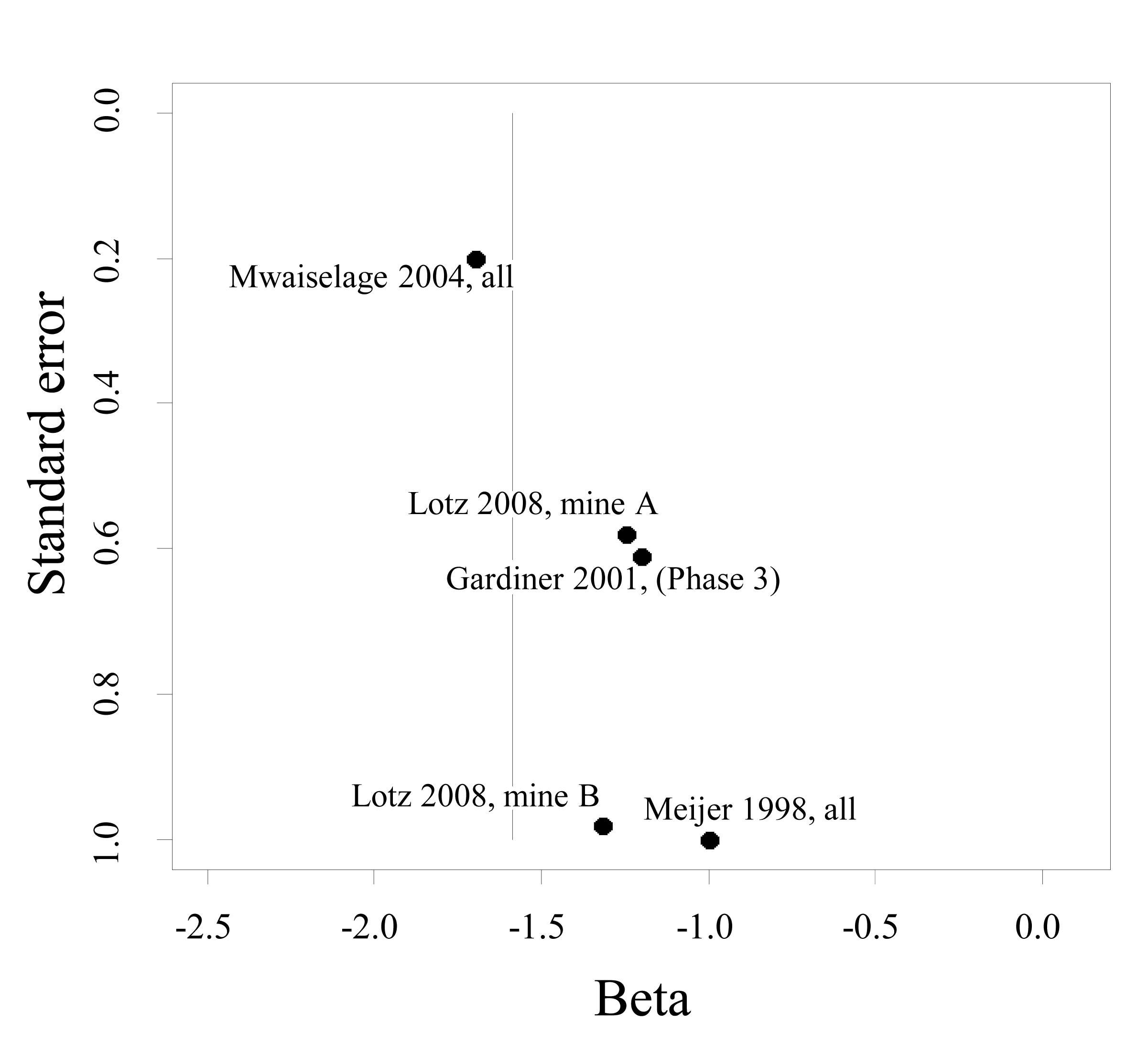
**

**Figure S4 Funnelplot for figure 5** Decrease of FEV1 (ml) in relation to the cumulative exposure to bg dust at the workplace (1 mg·m^-3^·years)

Table S1 Review of the 27 selected studies on biopersistent granular dust and airway obstruction by first author

| **First author/ year** | **Country** | **Bg dust exposure** | | **Industry/ measurements of exposure to inhalable and respirable dust [mg·m^-^³]** | **parameter chosen for meta-analysis (MA)** |
| --- | --- | --- | --- | --- | --- |
|  |  | yes | no |  |  |
| Abrons 1988 | USA | 2607 | 729 | Portland cement/ inhalable dust (GM*) 2.9 [mg·m^-^³], respirable dust (GM) 0.57 [mg·m^-^³] high vs. zero | FEV1 |
| [AbuDhaise 1997](#_ENREF_2) | Jordan | 99 | 129 | Portland cement/ 3 levels of exposure to respirable dust (GM) 0.5/1.6/3.9 [mg·m^-^³] (high vs. low exposure) | FEV1, FEV1/FVC |
| [Beach 2001](#_ENREF_6) | Australia | 572 | 79 | Bauxite open pit mining/ inhalable dust (GM) 0.44 – 0.65 mg·m^-^³, respirable dust (GM) 0.14-0.26 mg·m^-^³, Quartiles <2.5 /2.5-6.0/ 6.1-10/>10 mg·m^-^³·years | FEV1, FEV1/FVC – without SE, not suited for MA |
| Boojar 2002 | Iran | 141 | 65 | Manganese underground mining/ total dust (manganese content), cumulative respirable dust (JEM) [mg·m^-^³·years] | FEV1, FEV1/FVC |
| Chan-Yeung 1989 | Canada | 164 | 308 | aluminium smelter ‚High‘, if >50% working hours in the potroom | FEV1 |
|  |  | 75 | 115 |  | Decrease of FEV1 [ml ·year^-1^] |
| [Chen 2006](#_ENREF_10) | Taiwan | 394 | 309 | Steelworkers/ inhalable dust (AM) 3.55 mg·m^-^³, follow-up 1.90 mg·m^-^³ | FEV1, FEV1/FVC |
| [Fell 2003](#_ENREF_11) | Norway | 119 | 50 | Portland cement | Part of [Nordby 2011](#_ENREF_34) not included in MA |
| [Fine 1976](#_ENREF_14); Teil III | USA | 65 | 141 | Rubber workers/ respirable dust 1.05-3.00 mg·m^-3^ high vs. zero (cumulative dust years) | FEV1, FEV1/FVC |
| [Fine 1976](#_ENREF_13); Teil IV | USA | 91 | 141 | Talc workers/ respirable dust 0.47-3.55 mg·m^-3^ high vs. zero (cumulative dust years) |  |
| [Gardiner 1993](#_ENREF_15) | Europe | 509 | 277 | Carbon black/ inhalable dust max. 1.60 mg·m^-3^ and respirable dust >0.45 mg·m^-3^ in 5 exposure groups, JEM cumulative exposure [mg·m^-^³·months] | FEV1 |
| [Gardiner 2001](#_ENREF_16) | Europe | Phase 2: 2324 | Phase 3: 1994 | Phase 2, cumulative 263.2 mg·m^-^³·months; current exposure 0.77 mg·m^-^³ Phase 3, cumulative 245.9 mg·m^-^³· months; current exposure 0.57 mg·m^-^³ | Decrease of FEV1, FVC, FEV1/FVC per 1 mg·m^-3^ |
| [Harber 2003](#_ENREF_18) | USA | 416 | 236 | Carbon black / total, inhalable, and respirable dust, current and cumulative (JEM), classification into pentile groups | FEV1, decrease of FEV1 per mg·m^-3^·years without SE, not suited for MA |
| [Huvinen 1996](#_ENREF_20) | Finland | 36 | 93 | Stainless steel production/ Cr^+3^, Fe^+2^Cr_2_O_4_ (Chromit), „average dust concentration“ 1 – 1.8 mg·m^-3^ | FEV1, FEV1/FVC |
| [Johnsen 2008](#_ENREF_23) | Norway | 1812 | 532 | Smelter / inhalable dust and respirable dust according to working area | FEV1 |
| [Kongerud 1990](#_ENREF_25) | Norway | 1760 | 0 | Aluminium potroom workers/ total dust (median) 3.25 mg ·m^-^³ ; OR for obstruction according to duration of employment | FEV1 minus predicted divided by residual standard deviation – not suited for MA |
| [Kuo 1999](#_ENREF_26) | Taiwan | 291 | 105 | Foundry workers/ respirable dust 1.89 mg·m^-3^ (molding), 2.76 mg·m^-3^ (furnace), 2.07 mg·m^-3^ (after- processing) | FEV1, FEV1/FVC, |
|  |  | 308 | 112 |  | decrease of FEV1 [ml·year^-1^] without SE, not suited for MA |
| [Lotz 2008](#_ENREF_27) | Germany | 1.Study  A: 402  B: 438 | 0 | Underground potash mining Company A: respirable dust (AM) 1.96 mg·m^-^³ and inhalable dust (AM) 14.2 mg·m^-^³ ; cumulative respirable dust 613 mg·m^-^³·months; cumulative inhalable dust 4419 mg·m^-^³·months; Company B: respirable dust (AM) 0.88 mg·m^-^³ and inhalable dust (AM) 5.65 mg·m^-^³ ; cumulative respirable dust 165 mg·m^-^³·months, cumulative inhalable dust 1060 mg·m^-^³·months |  |
|  |  | 2.Study  A: 290  B: 278 | 0 |  | Decrease of FEV1 per 1·mg·m^-^³ |
| [Meijer 1998](#_ENREF_28) | Netherland | 70 | 69 | Rubber workers/ inhalable dust (AM) 2 mg·m^-^³ cumulative (JEM) 32.5 mg·m^-^³·years | FEV1, FEV1/FVC |
|  |  | 70 | 69 |  | decrease of FEV1 per mg·m^-3^·year |
| [Mwaiselage 2004](#_ENREF_31) | Tanzania | 115 | 102 | Portland cement/ inhalable dust (GM) 10.6 mg·m^-^³, cumulative dust (GM) 69.1 mg·m^-^³·years (high vs. low exposure) | FEV1, FEV1/FVC |
|  |  | 115 | 102 |  | decrease of FEV1 per 1 mg·m^-3^·year |
| [Neghab 2007](#_ENREF_32) | Iran | 88 | 80 | Portland cement/ inhalable dust (AM**) 53.4 mg·m^-^³ respirable dust (AM) 26 mg·m^-^³ | FEV1, FEV1/FVC |
| [Neghab 2007](#_ENREF_33) | Iran | 97 | 110 | Rubber industry/ inhalable dust (AM) 41.8 mg·m^-^³ and respirable dust (AM) 19.8 mg·m^-^³ | FEV1, FEV1/FVC |
| [Nordby 2011](#_ENREF_34) | Europe | 1406 | 629 | Portland cement/ inhalable dust (GM) 0.85 mg·m^-^³, classification by means of a JEM into quartiles <0.49/0.49—1.08/1.09—1.73/>1.74 [mg·m^-3^ ] | FEV1, FEV1/FVC and OR FEV1/FVC < 70% per 1 mg·m^-3^ inhalable dust |
| Selden 2001 | Sweden | 34 | 61 | Dolomite mining/ total dust (median): 2.8 mg·m^-3^ | FEV1 |
| [Soyseth 2011](#_ENREF_40) | Norway | 3392 | 532 | Smelter/ inhalable dust and respirable dust according to working area | OR for FEV1/FVC <70% per 1 mg·m^-3^ |
| [Townsend 1985](#_ENREF_42) | USA | 1146 | 0 | Aluminium production/ cumulative total dust (JEM) [mg·m^-3^ years] comparing < 100 mg·m^-3^ years and ≥ 100 mg·m^-3^ ·years for three categories of duration <10 years, 10-19 years, ≥ 20 years | FEV1 minus KNUDSON predicted FEV1 – not suited for MA |
| Wang 1996 | USA | 475 | 0 | Steel workers No dust measurements, exposed years in „dusty areas“ | Number of exposed years only, -not suited for MA |
| [Wild 1995](#_ENREF_44) | France | 138 | 55 | Talc producing/ respirable dust (GM) 1.87 mg·m^-^³ cumulative exposure according to JEM mg·m^-^³·years | Standardized residuals for FVC and FEV1 – not suited for MA |

For the studies with two rows, the 1st row is related to the cross-sectional analysis and the 2nd row to the longitudinal analysis

*GM: geometric mean

**AM: arithmetic mean

***JEM: job-exposure matrix

| **Section/topic** | **#** | **Checklist item** | **Reported on page #** |
| --- | --- | --- | --- |
| **TITLE** | | |  |
| Title | 1 | Identify the report as a systematic review, meta-analysis, or both. | title page |
| **ABSTRACT** | | |  |
| Structured summary | 2 | Provide a structured summary including, as applicable: background; objectives; data sources; study eligibility criteria, participants, and interventions; study appraisal and synthesis methods; results; limitations; conclusions and implications of key findings; systematic review registration number. | abstract |
| **INTRODUCTION** | | |  |
| Rationale | 3 | Describe the rationale for the review in the context of what is already known. | background |
| Objectives | 4 | Provide an explicit statement of questions being addressed with reference to participants, interventions, comparisons, outcomes, and study design (PICOS). | materials and methods |
| **METHODS** | | |  |
| Protocol and registration | 5 | Indicate if a review protocol exists, if and where it can be accessed (e.g., Web address), and, if available, provide registration information including registration number. | no |
| Eligibility criteria | 6 | Specify study characteristics (e.g., PICOS, length of follow-up) and report characteristics (e.g., years considered, language, publication status) used as criteria for eligibility, giving rationale. | materials and methods |
| Information sources | 7 | Describe all information sources (e.g., databases with dates of coverage, contact with study authors to identify additional studies) in the search and date last searched. | materials and methods |
| Search | 8 | Present full electronic search strategy for at least one database, including any limits used, such that it could be repeated. | materials and methods |
| Study selection | 9 | State the process for selecting studies (i.e., screening, eligibility, included in systematic review, and, if applicable, included in the meta-analysis). | materials and methods |
| Data collection process | 10 | Describe method of data extraction from reports (e.g., piloted forms, independently, in duplicate) and any processes for obtaining and confirming data from investigators. | materials and methods |
| Data items | 11 | List and define all variables for which data were sought (e.g., PICOS, funding sources) and any assumptions and simplifications made. | materials and methods |
| Risk of bias in individual studies | 12 | Describe methods used for assessing risk of bias of individual studies (including specification of whether this was done at the study or outcome level), and how this information is to be used in any data synthesis. | materials and methods |
| Summary measures | 13 | State the principal summary measures (e.g., risk ratio, difference in means). | statistical methods |
| Synthesis of results | 14 | Describe the methods of handling data and combining results of studies, if done, including measures of consistency (e.g., I^2^) for each meta-analysis. | statistical methods |

Page 1 of 2

| **Section/topic** | **#** | **Checklist item** | **Reported on page #** |
| --- | --- | --- | --- |
| Risk of bias across studies | 15 | Specify any assessment of risk of bias that may affect the cumulative evidence (e.g., publication bias, selective reporting within studies). | statistical methods |
| Additional analyses | 16 | Describe methods of additional analyses (e.g., sensitivity or subgroup analyses, meta-regression), if done, indicating which were pre-specified. | statistical methods |
| **RESULTS** | | |  |
| Study selection | 17 | Give numbers of studies screened, assessed for eligibility, and included in the review, with reasons for exclusions at each stage, ideally with a flow diagram. | results |
| Study characteristics | 18 | For each study, present characteristics for which data were extracted (e.g., study size, PICOS, follow-up period) and provide the citations. | results |
| Risk of bias within studies | 19 | Present data on risk of bias of each study and, if available, any outcome level assessment (see item 12). | no |
| Results of individual studies | 20 | For all outcomes considered (benefits or harms), present, for each study: (a) simple summary data for each intervention group (b) effect estimates and confidence intervals, ideally with a forest plot. | results |
| Synthesis of results | 21 | Present results of each meta-analysis done, including confidence intervals and measures of consistency. | results |
| Risk of bias across studies | 22 | Present results of any assessment of risk of bias across studies (see Item 15). | funnelplots |
| Additional analysis | 23 | Give results of additional analyses, if done (e.g., sensitivity or subgroup analyses, meta-regression [see Item 16]). | results |
| **DISCUSSION** | | |  |
| Summary of evidence | 24 | Summarize the main findings including the strength of evidence for each main outcome; consider their relevance to key groups (e.g., healthcare providers, users, and policy makers). | discussion |
| Limitations | 25 | Discuss limitations at study and outcome level (e.g., risk of bias), and at review-level (e.g., incomplete retrieval of identified research, reporting bias). | discussion |
| Conclusions | 26 | Provide a general interpretation of the results in the context of other evidence, and implications for future research. | discussion |
| **FUNDING** | | |  |
| Funding | 27 | Describe sources of funding for the systematic review and other support (e.g., supply of data); role of funders for the systematic review. | online submission system |

*From:*  Moher D, Liberati A, Tetzlaff J, Altman DG, The PRISMA Group (2009). Preferred Reporting Items for Systematic Reviews and Meta-Analyses: The PRISMA Statement. PLoS Med 6(6): e1000097. doi:10.1371/journal.pmed1000097

For more information, visit: **www.prisma-statement.org**.

Page 2 of 2
